# Biophysics involved in the process of tumor immune escape

**DOI:** 10.1016/j.isci.2022.104124

**Published:** 2022-03-19

**Authors:** Maonan Wang, Hui Jiang, Xiaohui Liu, Xuemei Wang

**Affiliations:** 1 State Key Laboratory of Bioelectronics (Chien-Shiung Wu Lab), School of Biological Science and Medical Engineering, Southeast University, Nanjing 210096, China

**Keywords:** Biological sciences, Immunology, Biophysics

## Abstract

Much of the current research into immune escape from cancer is focused on molecular and cellular biology, an area of biophysics that is easily overlooked. A large number of immune drugs entering the clinic are not effective for all patients. Apart from the molecular heterogeneity of tumors, the biggest reason for this may be that knowledge of biophysics has not been considered, and therefore an exploration of biophysics may help to address this challenge. To help researchers better investigate the relationship between tumor immune escape and biophysics, this paper provides a brief overview on recent advances and challenges of the biophysical factors and strategies by which tumors acquire immune escape and a comprehensive analysis of the relevant forces acting on tumor cells during immune escape. These include tumor and stromal stiffness, fluid interstitial pressure, shear stress, and viscoelasticity. In addition, advances in biophysics cannot be made without the development of detection tools, and this paper also provides a comprehensive summary of the important detection tools available at this stage in the field of biophysics.

## Graphical abstract

**Figure:**
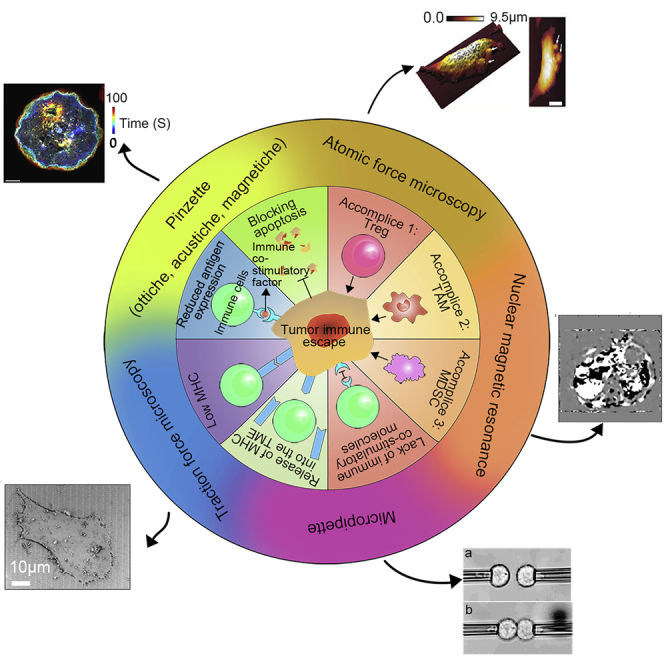

## Introduction

The recently published summary on fourteen characteristics of tumors has renewed the interest of researchers in the molecular and cellular biology of tumors (Hanahan 2022). It is undeniable that molecular biology and cell biology have made significant contributions to tumor research (Cullin et al., 2021; Kimmelman and White 2017; Morad et al., 2021; Liu et al., 2021a, 2021b, 2021c; Certo et al., 2021; Pardi et al., 2018). However, in recent years, the outstanding contribution of biophysics in tumor research has also received wider attention as research has progressed. Biophysics applies the traditional concepts of physics to the study of biological and physiological problems, linking living phenomena to physical states, breaking down the barriers between disciplines, and greatly facilitating scientific progress (Moriarty et al., 2022). The study of the stiffness and mechanical response of tumor cells and the related mechanical processes that take place with the tumor microenvironment through the blood flow of the vascular system is very important for the study of tumor killing. It has been found that the mechanism of movement of cancer cells differs from that of normal cells and that the absence of internal scaffolding proteins alters the mechanical stiffness of the cells, making them more susceptible to deformation (Bao and Suresh 2003; Zhu et al., 2000; Janmey et al., 1991). The molecular structure and expression of cytoskeletal proteins determines the mechanical microenvironment between the cell and adjacent cells. F-actin keeps the cell in place when subjected to high stress. F-actin provides resistance to deformation without causing the cell to become a “shear-thinning fluid” under high shear stress (Janmey et al., 1990; O'Melia et al., 2019). The biophysical changes involved in tumor invasion have been fully addressed (Suresh, 2007). However, the biophysical changes involved in tumor immune escape have not been adequately elucidated.

Cancer immunology came into the limelight at this time when James P. Allison and Tasuku Honjo were awarded the Nobel Prize in Physiology or Medicine for their discovery of the treatment of cancer through the suppression of negative immune regulation (Altmann 2018). Tumor immune escape is an important factor in the development of tumors (Borcoman et al., 2022). The immune system guards the health of the body, and therefore tumor cells can only proliferate and metastasize if they escape the surveillance and attack of the immune system. Tumor immune escape underpins other features of tumor cells. Immunotherapy is one of the important adjuvant therapies for anti-tumor treatment. In 2021, the first CAR T-cell therapy was approved by the Chinese State Drug Administration. “CAR-T” therapy stands for “Chimeric Antigen Receptor T-cell Immunotherapy,” and it works as an immunotherapy by remodeling the patient’s T cells *in vitro* and then placing the remodeled T cells into the patient’s body, thereby blocking the patient’s immune escape (Maganti et al., 2022). It can be seen that research based on tumor immune escape can have important implications for understanding the pathogenesis of tumors or developing drugs.

This review concisely reviews the mechanisms by which tumor immune escape occurs and summarizes the biophysical concepts involved in the current phase of research into this process. Biomechanics is one of the larger branches of biophysics. The study of tumor-related biomechanics is more common. The focus here is concentrated on recent advancements and challenges of the biophysical studies concerning mechanical stress and other relevant factors. It also describes the methods commonly used at this stage to detect mechanical stress in tumors, which are designed to accelerate the study of tumors through a biophysical perspective.

## Mechanisms of tumor immune escape

**Figure 1:**
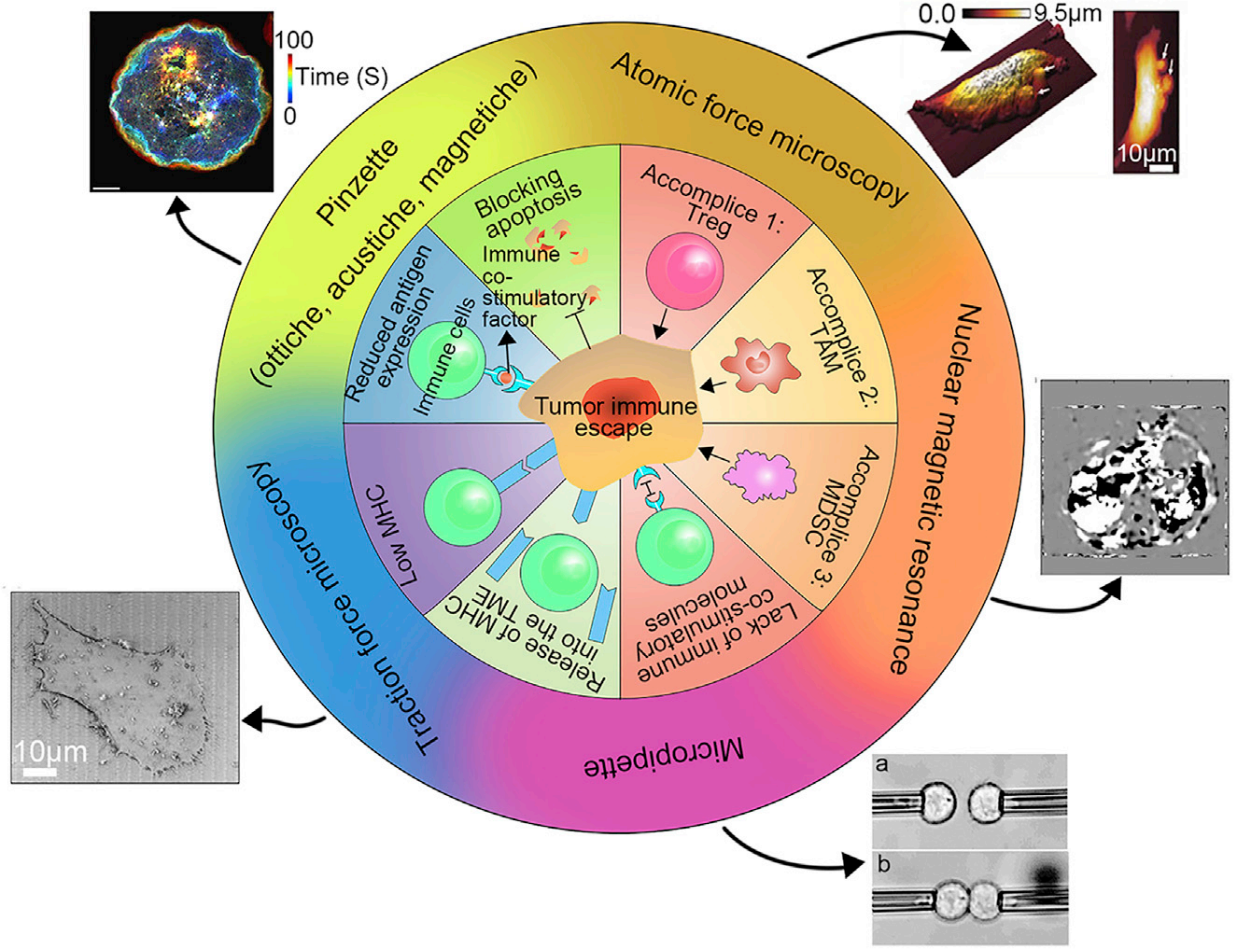
Two kinds of ways for tumor cells to acquire immune escape and tools needed to detect this immune escape process The first is to directly acquire escape properties through the self-regulation of cancer cells; the second method is the indirect way. By reeducating the immune cells, the surveillance of the immune cells is loosened. TAM, Tumor-associated macrophages; MDSC, Myeloid-derived suppressor cells; TME, Tumor immune microenvironment. The outermost circle shows common tools for biophysical assays, as well as demonstration images of the different tools, from each of these high-level papers: Efimova et al., (2020). Copyright CC BY-NC 4.0; Hu and Shan (2020). Copyright. © 2020 Elsevier B.V.; Dufour et al., (2005). Copyright 2022, American Physical Society; Park et al., (2017). Copyright CC BY license; Li et al. (2021a, 2021b). Copyright CC BY4.0.

In the 1970s, Burnet et al. proposed the theory of immune surveillance, arguing that the immune system can monitor mutant cells that “do not belong to itself”, and can remove these mutant cells in a targeted manner through cellular immune mechanisms to maintain the body’s internal environment. However, when the mutant cells escape the surveillance of the immune system under the action of various factors, they will rapidly proliferate in the body and accelerate the deterioration of the tumor. This is the concept of tumor immune escape (Wang et al., 2021). The study found that tumors mainly achieve immune escape in two ways: one is to directly acquire escape properties through the self-regulation of cancer cells, and the other is to indirectly relax the monitoring of mutated cells by immune cells. Here we briefly describe these two immune escape pathways (Figure 1).

Tumor cells can acquire immune escape directly through five pathways. The first is that tumors escape immune escape by preventing their own apoptosis (Song et al., 2022; Li et al., 2021a, 2021b). The first step in generating tumor immunity is thought to be the migration of dendritic cells (DCs) toward the apoptotic tumor mediated by various chemokines. In a mouse model of tumor treatment with ganciclovir, ganciclovir was found to induce not only apoptosis but also infiltration of DCs within the tumor (Iida et al., 2008). In addition, tumor cells lacking the HOIP gene were highly sensitive to NK and CD8^+^ T cell-mediated killing. Moreover, deletion of the HOIP gene can promote apoptosis (Freeman et al., 2021). The second modality is the reduced expression of antigens on the surface of tumor cells. The surface tumor antigen family of T cells has been found to contain more than 250 members. As early as 2009, it was discovered that the expression of the corresponding T cell receptors by genetic engineering could directly target cancer cells expressing the corresponding antigens and produce killing effects to eliminate tumors (Johnson et al., 2009; Waldman et al., 2020). It is worth mentioning that the gradual diminution of antigen expression in tumor cells appears to be an evolutionary process that is constantly being screened. Because of the heterogeneity of tumor cells, different tumor cells in different parts of the same organism have different types and levels of antigen expression, and different tumor cells in the same part of the same organism have different types and levels of antigen expression. Tumor cells with more antigen types and higher expression levels will be preferentially recognized and eliminated by the immune system. After being eliminated by the immune system, tumor cells with relatively weak immunogenicity can escape the surveillance of the immune system and thus can proliferate. As a result, the antigen expression of tumor cells becomes weaker and weaker.

A third way is the low expression of MHC molecules by tumor cells. The phenomenon of 'MHC restriction' was first highlighted by Doherty and Zinkernagel in 1975 when they studied the viral immune response in mice. MHC restriction, whereby viral peptides are only recognized by T cells when bound to specific MHC molecules, was demonstrated (Murray et al., 2016). The lack of MHC class I molecules is often one of the main causes of immune escape from tumors. The expression of MHC class I molecules varies between tumor cells, with MHC I being weakly expressed in poorly differentiated tumor cells and weakest or even absent in metastatic tumors. In addition, most solid tumors do not express MHC class II molecules and do not effectively activate T helper cells (Deng et al., 2007; Brentville et al., 2016). The fourth pathway is the release of histocompatibility complex MHC molecules (Westwood et al., 2009). Tumor cells can release soluble antigen molecules and these free tumor antigens bind to antitumor antibodies to form complexes that can bind to the Fc receptors of immune cells such as NK cells via the Fc segment of the antibody. This depletion of antitumor antibodies and the shutting down of Fc receptors prevents immune cells from performing their normal role of tumor destruction (Pahl and Cerwenka 2017; Nigro et al., 2019; Nimmerjahn and Ravetch 2007; Ochoa et al., 2017). Although it is not clear by what mechanism tumor cells release MHC molecules, high expression of MHC in the body fluids surrounding tumor cells does deplete immune killer cells. The fifth pathway is the lack of co-stimulatory molecules in tumor cells (Guerra et al., 2022). It has been found that tumor cells express antigen and antigen presenting molecules; however, in the absence of co-stimulatory molecules, they are still unable to activate T cells for immune killing. Such molecules include, for example, ICAM-I (Chiriva-Internati et al., 2006), IFA-3 (Rahimian et al., 2015), VCAM-1 (Paessens et al., 2008), TNFR(Croft 2003), or OX40 (Fu et al., 2020). Indirect relaxation of immune cell regulatory pathways involves the reeducation of the immune system by tumor cells. The current study found that regulatory T cells (Tregs), tumor-associated macrophages (TAM), and myeloid-derived suppressor cells (MDSC) among immune cells can help tumor cells evade immune surveillance. Tregs are a heterogeneous population consisting mainly of CD4^+^ CD25h/Treg, Trl (IL-10 + T cells), and TGF-β+ T cells. Tregs have the ability to promote tumor development by inhibiting the activity of most other lymphocytes and dendritic cells through a mechanism of intercellular contact (Raghavan and Quiding-Jarbrink 2011; Glasner and Plitas 2021). In addition, TAM has been found to play an “accomplice” role in the development and metastasis of tumors, such as liver, lung, and breast cancers. TAM not only suppresses antitumor immunity but also expresses and secretes pro-inflammatory and pro-oncogenic factors in large amounts, directly promoting tumor growth, metastasis, and invasion. Many studies have shown that when macrophage antagonists are combined with ICB therapy, a good prognosis is often achieved (Ruffell and Coussens 2015; Zhu et al., 2014). Another immune cell accomplice in tumor development is MDSC, which expresses a variety of pro-angiogenic factors that directly promote tumor angiogenesis while suppressing T cell-mediated adaptive antitumor immunity as well as NK and macrophage-mediated natural antitumor immunity through high expression of ARG1, iNOS, and ROS (Draghiciu et al., 2015; Redd et al., 2017; Adeshakin et al., 2021).

## Biomechanics on tumor cells during immune escape

Tumor cells are inevitably subject to forces in the body. Biomechanics is an important branch of biophysics. The acquisition of the aforementioned tumor escape characteristics by tumor cells is inextricably linked to biomechanics, and the biomechanically relevant concepts involved in tumor cells are summarized in Table 1. Tumor cells regulate and sense the mechanical features of the tumor surroundings and translate these mechanical force signals into biochemical signals inside and outside the cell through transmembrane proteins with cytoskeletal proteins, mucins, integrins, etc., which in turn modify their own behavior and even the biological signals affecting other surrounding cells. Therefore, it is important to study the biomechanical information involved in the process of tumor immune escape.

**Table 1:** Biomechanical concepts involved in tumor immune evasion

Name	Origins	Physical explanation	Interacting parties in tumor immune escape	Ref
Stress	Tumor and stromal hardness	When an object is deformed because of an external cause, an internal force is generated between the parts of the object that interact to resist the action of this external cause	Tumor cells and tumor cells; Tumor cells and tumor stromal cells; Tumor cells and mesenchyme	(Levinson et al., 2008; Vitale et al., 2019; Xin et al., 2020; Mina et al., 2017)
Shear stress	Blood flow	The cross-section between the two forces is the dividing line, along which the two parts of the member become relatively misaligned. This form of deformation of the member is known as shear, and its cross section is the shear surface. The magnitude of the shear force per unit area of the cross section is called shear stress	Blood flow and peripheral cells	(Maeshiro et al., 2021; Pomianek et al., 1996)
Strain	∖	Describes the deformation of an object relative to its original length, measured as a percentage	Solid tumor; intravascular wall	(Cardillo et al., 2006)
Stiffness	∖	Describes the elasticity of a material or the recovery of its original shape after deformation	Solid tumors; fibroblasts; extracellular matrix	(Seewaldt 2014; Fenner et al., 2014; McLane and Ligon 2015)
Flexibility	∖	Describes the ability of an object to recover its original shape after the removal of an external force.	Solid tumors; intravascular walls; extracellular matrix	(Szabo and Merks 2013)
Viscoelasticity	∖	Objects exhibit both elastic and viscous properties when they deformed. The strain of a viscous material is time-dependent, whereas an elastic material is time-independent	Cells with adjacent cells	(Mierke 2021)

### Tumor and stromal stiffness promote the process of tumor immune escape

**Figure 2:**
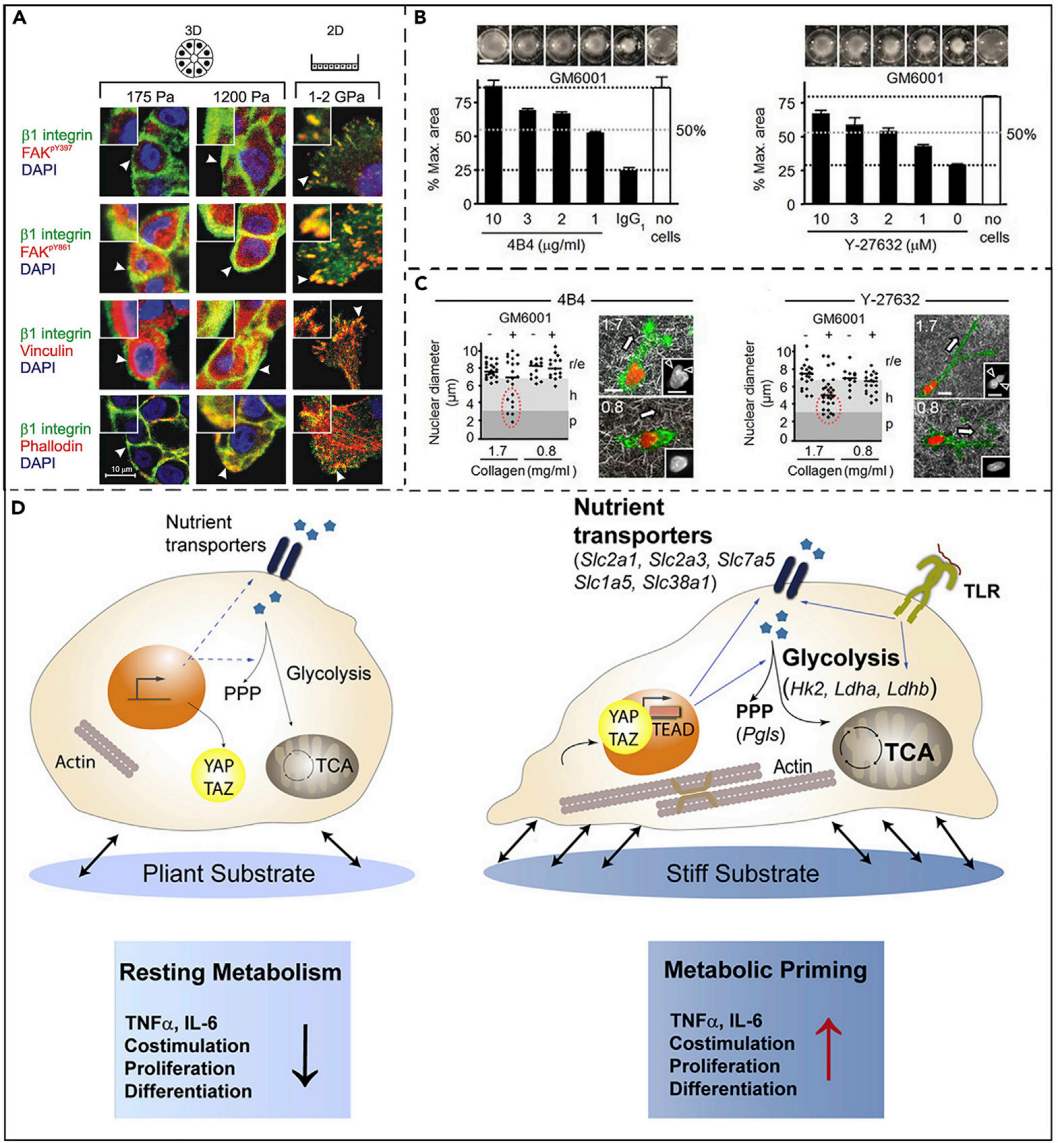
Larger stiffness of tumor and stroma compared to normal cells (A) Confocal fluorescence images of β1 integrin adhesion (green) in MEC in 3D (soft vs. hard) and 2D (hard) BM/COL I gel at different pressures of 175 and 1200 Pa. The proteins represented in red vs. blue are identified on the left side of the image (Paszek et al., 2005). Copyright © 2005 Elsevier. (B) Inhibition of collagen contraction by anti-integrin type 1 mAb 4B4 (left) or Y-27632 (right), which also dose-dependently inhibits collagen contraction (Wolf et al., 2013). Copyright 2013, Rockefeller University Press. (C) Size analysis of the nuclei of different cells at different collagen concentrations (Wolf et al., 2013). Copyright 2013, Rockefeller University Press. (D) Mechanically stiff mechanisms could activate the Hippo signaling molecule TAZ as well as Ca^2+^-associated ion channels, including potentially PIEZO1, which can influence dendritic cell metabolism to trigger adaptive immune responses (Chakraborty et al., 2021). Copyright Creative Commons CC-BY-NC-ND license.

It is well-known that tumors are stiffer than normal tissue because of the high fibrosis at the tumor tissue, the stiff stroma of the tumor cells, and the elevated expression of cytoskeleton-related proteins (Beshay et al., 2022). Correlation stiffness maps of biopsies obtained from different locations of breast tumors show a progressive increase in extracellular matrix (ECM) stiffness from the center to the periphery (Paszek et al., 2005) (Figure 2A). Although there is no clinical evidence that hardness heterogeneity within the tumor is positively associated with a poorer prognosis for patients with tumors, yet there has been evidence that tumor invasion and poorer patient prognosis are associated with a higher probability of tissue fibrosis and a stiffer stroma (De Marco et al., 2022). Tumor stiffness reflects increased interstitial tissue pressure, solid stress, and elastic modulus (Paszek et al., 2005). In terms of tumor immune escape, tumor stiffness is inextricably linked to this (Chirivì et al., 2021; De Marco et al., 2022). In the tumor microenvironment, fibroblasts, which maintain tumor stiffness, can secrete factors such as CXCL12 to inhibit the movement of T cells toward the tumor. Results based on mouse models show that genetic engineering to ablate tumor-associated fibroblasts can activate antitumor immunity and enhance the effectiveness of immunotherapy (Feig et al., 2013; Kraman et al., 2010). In addition, the increased ECM of tumor tissue produces a dense collagen layer, a physical barrier that attenuates the movement of immune cells deeper into the tumor (Wolf et al., 2013). Matrix metalloproteinases, integrins, and myosin in the ECM have been found to mediate the mechanical coupling of immune cells moving deeper into the tumor. How these parameters work together, however, remains unclear (Figures 2B and 2C).

However, it was certain that when T cells were exposed to high concentrations of mouse collagen mimics *in vitro*, their exercise capacity was greatly reduced (Wolf et al., 2013). At the same time, the mechanical stiffness of the tumor cell machinery regulates dendritic cell metabolism, with increased glucose metabolic flux in dendritic cells grown at higher stiffness, while directing human monocytes to derive a DC phenotype (Chakraborty et al., 2021) (Figure 2D). Specifically, the expression of Wwtr1 (TAZ) gene was significantly upregulated in fresh dendritic cells isolated on 50 kPa hydrogel compared with fresh dendritic cells isolated on 2 kPa hydrogel; therefore, TAZ can enter the nucleus more easily under mechanical stress and exert transcriptional regulatory effects on downstream tumor-promoting genes. In turn, decreased glucose uptake and increased dendritic cell proliferation were observed in TAZ knockout mice. TAZ appears to link the rigidity of tumor cells to the glucose metabolism function of dendritic cells. In general, it is very important to study the relationship between the hardness of tumor cells and matrix and immune cell metabolism and immune microenvironment remodeling (Chirivì et al., 2021). A study by Rizzi’s team last year quantified the effect of tumor matrix stiffness on regulatory T lymphocytes in three ways (Chirivì et al., 2021). First, when T cells are in a three-dimensional state, the pressure on the cell membrane is distributed throughout the cell, the cell suddenly becomes smaller, and the cell volume decreases significantly. Second, the nucleus of T cells subjected to 360° circumferential pressure becomes smaller, which may be a deformation caused by the response of T cells to circumferential pressure (González-Bermúdez et al., 2020). The third aspect is that high-density ECM is also selective to the change of T cells. It is found that high-density ECM is conducive to the activity of CD4 ^+^ T cells rather than CD8 ^+^ T cells. This is achieved by changing the gene expression of different T cells. CD4 ^+^ T cells promote the development of inflammation, whereas CD8 ^+^ T cells kill tumors (Durgeau et al., 2018).

### Fluid interstitial pressure promotes the process of tumor immune escape

The abundant and abnormal vascular system in the tumor creates a high interstitial fluid pressure environment for solid tumors. Compared with normal tissues, this high interstitial fluid pressure can promote the invasion and metastasis of cancer cells to surrounding tissues (Jain 2005; Raju et al., 2008). Interstitial fluid is composed of interstitial and solute, including intercellular protein and matrix. The pressure gradient of solid tumor promotes the flow of stroma and the growth of lymphatic vessels at the edge of tumor (Kim et al., 2009; Kataru et al., 2009; Flister et al., 2010). Lymphatic vessels provide a highway for tumor metastasis and immune escape.

**Figure 3:**
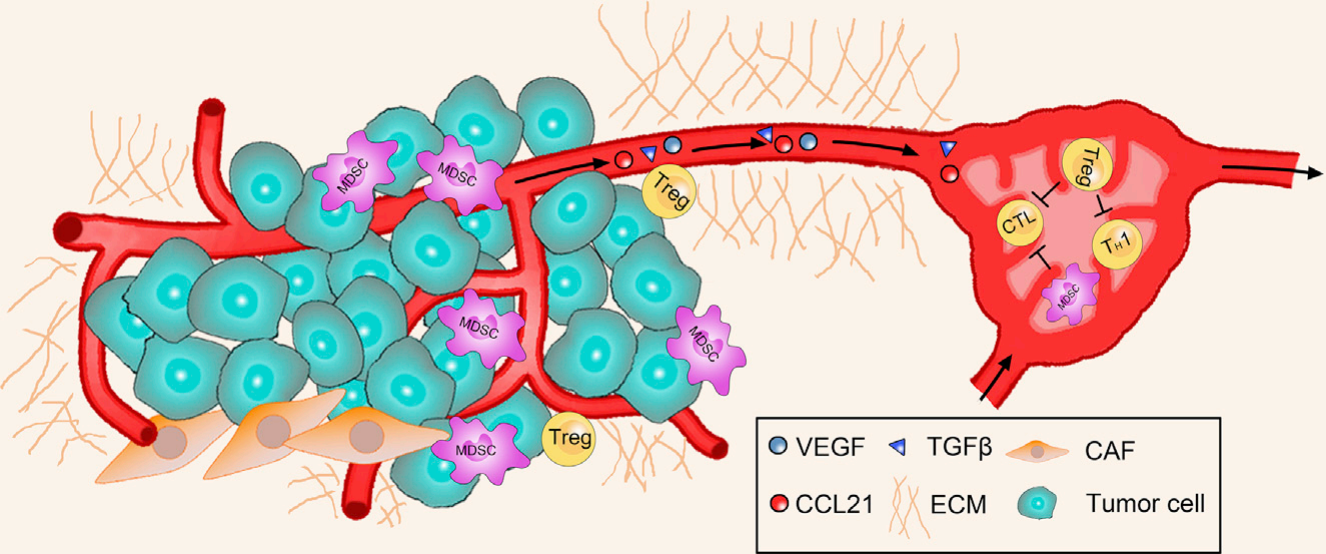
Lymphatic and interstitial pressure and shear stress in the tumor microenvironment prevent immune cells from entering the tumor site Stiffer tumor cells block immune cells from the outside of the tumor, and the pressure difference forces some lavage-free cell re-education factors into the lymph nodes.

One study measured a mean interstitial pressure (IFP) of 29.8 mmHg in 22 melanoma patients with tumor nodules (Curti et al., 1993). The mean tumor IFP of breast ductal carcinoma patients was 29 mmHg (Nathanson and Nelson 1994). In studies of xenogeneic pancreatic ductal carcinoma, the IFP range of the tumor was found to be 75–130 mmHg (Provenzano and Hingorani 2013). Although the flow kinetics of different tumor stroma may be different, the specific value remains to be further studied. But it is certain that a large number of cytokines, including TGFβ1, are squeezed into the surrounding tissue by gradient fluid pressure between the solid tumor and surrounding tissue and by stromal tension. TGFβ1 suppresses the immunotoxicity of T lymphocytes and helps tumor cells escape immunity. Interstitial pressure promotes the production of CCL21 cytokines in the tumor microenvironment, and high levels of CCL21 can alter the form of antigen presentation and inhibit the movement of immune cells to the tumor. A large number of tumor antigens and cytokines, driven by compression or rheology, flow to the lymph nodes, promoting tumor immune escape from the lymph node location (Swartz and Lund 2012) (Figure 3). At the same time, immature dendritic cells are exposed to tumor factors for a long time, which is not conducive to their development and maturation (Liu and Wang 2009). The macrophages summoned to destroy tumors are also more easily educated into tumor-related macrophages under the stimulation of tumor factors; therefore, they are also called accomplices of tumor immune escape (Solinas et al., 2009; Jiang et al., 2022). It is worth mentioning that tumor interstitial fluid dynamics also bring obstacles to tumor treatment, because it is difficult for exogenous drugs to reach the tumor site because of flow pressure. But the familiar EPR effect is closely associated with high interstitial fluid dynamics. The EPR effect exploits the high leakiness of the tumor vasculature to enhance drug retention at the tumor site (Iyer et al., 2006). Most nanomedicine targeting involves the principle of exploiting the EPR effect at the tumor site. However, the high interstitial fluid dynamics at the tumor site suggests that in the future, targeted drug design needs to first consider overcoming the high interstitial fluid dynamics at the tumor site, and then exploit the EPR effect for therapeutic purposes.

### Shear stress promotes the process of tumor immune escape

In the process of fluid flow, the stress on the surrounding medium is called shear stress. In a study conducted using the microfluidic platform in 2013, it was found that 3D ovarian cancer micronodules grown under continuous laminar flow showed elevated EGFR expression and enhanced cancer promoting phenotypes, such as EMT (Rizvi et al., 2013). The study found that cancer cells seem to be more likely to grow in locations with high shear stress. But in another study, it was found that cancer cells were more likely to extravasate at the bifurcation of veins with low shear stress. The rich and abnormal vascular system at the tumor cells hinders the high-speed flow of blood. At this time, the immune cells in the blood flow are recruited to the tumor site, which provides a fulcrum for the colonization of tumor cells in the blood. Cancer cells can rely on immune cells to escape the vascular system and enter the endothelial layer. For example, with the help of neutrophils, the extravasation efficiency of melanoma increased by 85%, and it can be colonized under the shear stress of 4 dyn/cm^2^ (Slattery and Dong 2003). Overall, this may mean that high shear force contributes to the malignant phenotype of tumor cells, but the colonization of tumor cells from the vascular system is because of low shear force (Badia-Ramentol et al., 2021). In addition, the mystery of the relationship between the action of shear stress and the protein will be gradually unraveled. For example, Liu’s team already found back in 2018 that shear stress promotes loss-of-nest apoptosis resistance in cancer cells through caveolin-1-dependent extrinsic and intrinsic apoptotic pathways (Li et al., 2018).

### Viscoelasticity promotes the process of tumor immune escape

It is found that the viscoelastic parameters of tumor cells are lower, and the cells are easier to deform and flow. Therefore, cell viscoelasticity can be used to distinguish tumor cells from normal cells (Nematbakhsh et al., 2017). The cells hardly move on the matrix with elastic modulus of 2 kPa but move very widely on the matrix with elastic modulus greater than 2 kPa and show rapid stress relaxation (Adebowale et al., 2021). Although the viscoelasticity of tumor cells is not easy to be quantitatively detected, it can be simulated with vertex model (Manning et al., 2010; Staple et al., 2010; Bi et al., 2014), Potts model (Vroomans et al., 2015), and topological model (Mongera et al., 2021). A large number of hydrogel materials can also mimic the matrix of tumor cells and adjust the viscoelasticity of hydrogel materials, which can mimic the viscoelasticity of tumor matrix (Mongera et al., 2021). *In vitro* studies have found that the relaxation of substrate materials will promote the diffusion speed of tumor cells and increase the diffusion area (Chaudhuri et al., 2015).

**Figure 4:**
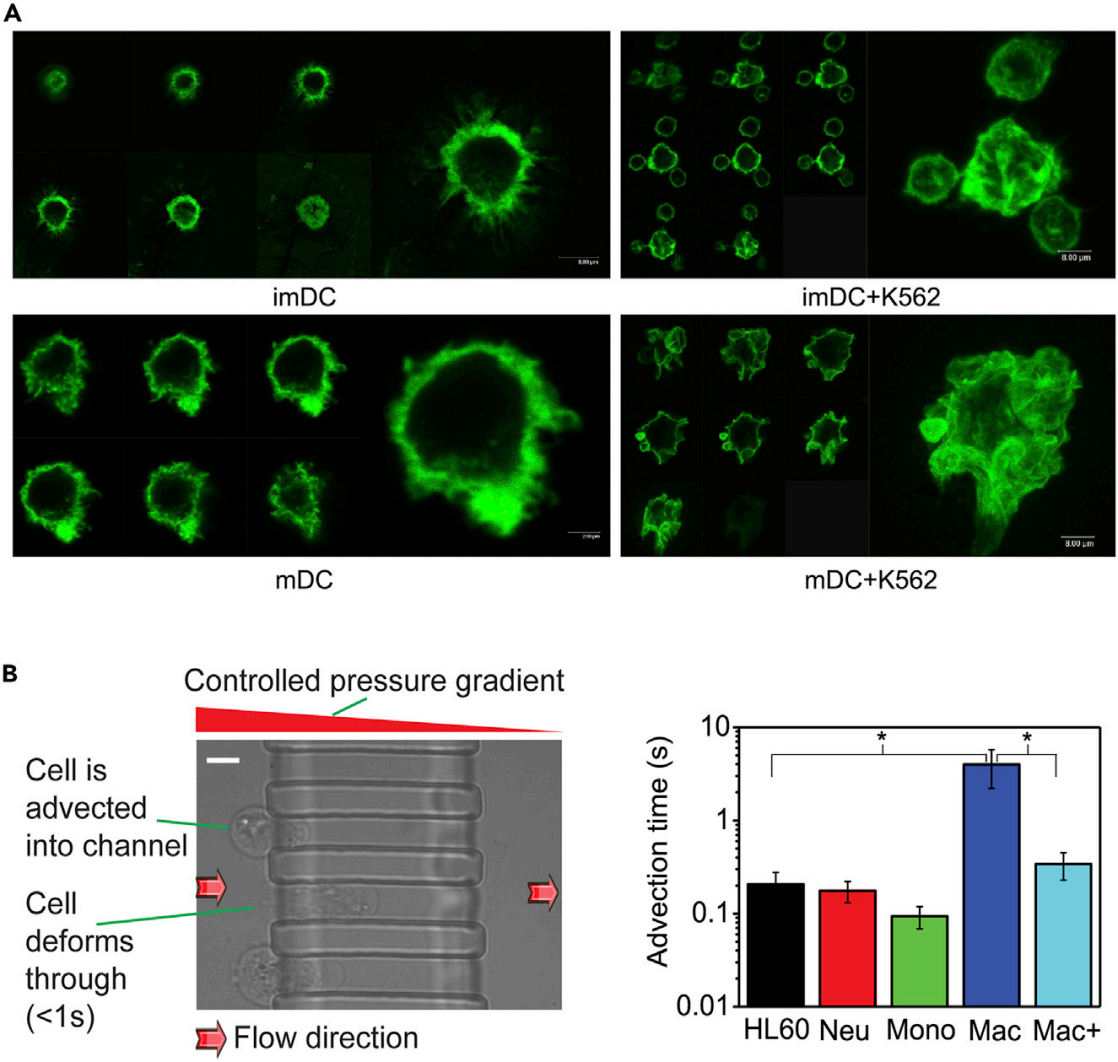
Analysis of viscoelasticity of immune cells (A) Representative series of high-resolution fluorescence confocal images of rhodamine-labeled F-actin in dendritic cells (including Immature dendritic cells (imDCs) and Mature dendritic cells (mDCs)) and DCs (imDCs + K562 and mDCs + K562) after K562 education in leukemic cells. Representative series of high-resolution fluorescence confocal images. This indicates that after cancer cells educate DC cells, DC cells are less viscoelastic and more susceptible to deformation (Xu et al., 2010). Copyright © 2010 Elsevier Ltd. (B) The time for cells to pass through the 12 × 12 μm channel shown on the left was recorded under a pressure of 20 mbar. It can be seen that the average time for macrophages (n = 50) is 3.98 ± 1.77 s, an order of magnitude longer than all other cell types, with a scale bar of 10 μm. This indicates that macrophages are more viscoelastic in nature (Ekpenyong et al., 2012). Copyright. CC-BY 4.0.

Changes in cellular viscoelasticity may have an early warning effect on disease (Harris et al., 2019). As a blood tumor, the pathogenesis of leukemia has always been one of the key research objects in the medical field. The study found that when a human chronic myeloid leukemia cell line was cocultured with dendritic cells, the viscoelasticity of dendritic cells and downstream signaling pathways were altered (Xu et al., 2010). Similar results were found in experiments with other tumor cells. Treatment of dendritic cells with cancer cells and cell cultures resulted in a severe deterioration of their biophysical properties, which greatly facilitated tumor immune evasion (Zeng et al., 2015). These findings show that the reeducation of immune cells by tumor cells not only reflected in the expression of some biological analysis signals but also in biophysics. Dendritic cells are responsible for antigen presentation and play a central role in the initiation, regulation, and maintenance of immune responses (Figure 4A). Vascular endothelial growth factor VEGF has been found to inhibit the immune function of dendritic cells. One of the inhibitory mechanisms is the remodeling of dendritic cell viscoelasticity (Xu et al., 2010). Blocking the transmission of VEGF is of great significance for restoring the physical properties of dendritic cells and remodeling the tumor immune barrier (Hu et al., 2016). Beyond that, one study even tracked immune cells and found that the viscoelastic properties of monocytes are conferred early in the differentiation of bone marrow precursor cells (Figure 4B). When HL60 cells were induced to differentiate along neutrophils, their deformability increased and their viscoelasticity decreased (Ekpenyong et al., 2012). Cell viscoelasticity may also serve as a therapeutic target for tumor migration and differentiation behavior.

### Effect of irradiation on biophysical parameters in tumor immune escape

There are many factors influencing the biomechanical parameters of tumor cells, and the emphasis here is on the radiation, as this is also a means of tumor treatment, and a thorough study of its impact on biophysics is even more significant for clinical treatment. Elucidation of the mechanisms of immune response under irradiation exposure, including biophysical mechanisms may allow early adjuvant therapy.

Already in 1970, an article published in Nature found that exposure to ionizing radiation affects the electrophoretic mobility of tumor cells (Repacholi 1970). Since then, a large number of researchers have invested in research on irradiation and tumor biophysics and have found that ionizing radiation can reduce the stiffness of isolated tumors and *in vitro* collagen matrices (Miller et al., 2018). Interstitial fluid pressure (IFP) has been shown to be an independent prognostic parameter for disease-free survival in patients with cervical cancer treated with radiation, as IFP correlates significantly with the proportion of acutely hypoxic cells (Rofstad et al., 2009). Interstitial fluid not only plays an important role in the delivery of anticancer agents but also modulates the sensitivity of hypoxic tumor cells to radiotherapy. Shear stress has been found to enhance radiation toxicity to colon cancer cells by inhibiting integrin signaling and FAX protein degradation (Luo et al., 2014). Taken together, it can be concluded that irradiation has a significant effect on the biomechanical parameters of tumor cells. Although there are no studies on the biophysical parameters involved in the immune escape process of tumors by irradiation, it has been found that local irradiation promotes tumor cell apoptosis, leading to the release of tumor antigen signals from solid tumors. These tumor apoptotic signals along with tumor antigens promote dendritic cell maturation, activating tumor specificity and leading to distal tumor regression (Mendes et al., 2016). This could explain the distal regression of tumor cells induced by local irradiation. But the mechanism in which immune surveillance is restored in humans is unclear and needs to be explored by studying the biophysics together with molecular biology of tumors.

## Biophysical detection methods involved in tumor immune escape

### Atomic force microscope

Atomic Force Microscopy (AFM), also known as Scanning Force Microscopy (SFM), is a nanoscale, high-resolution scanning probe microscope that is 1000 times above the optical diffraction limit. AFM is the most important tool for manipulating, imaging, and measuring materials at the nanoscale (Wang et al., 2020). Relevant information is gathered through microcantilever sensing and the “feeling” of the thin probe surface on the cantilever, whereas piezoelectric elements can control the sample or scanner for very precise and minute movements. The microcantilever senses and amplifies the force between the cantilever tip and the atoms of the sample under test to enable detection with atomic-level resolution (Horber and Miles 2003). The two main working modes of atomic force microscopes are static mode and dynamic mode (Zhang et al., 2010). In static mode, the cantilever traverses the surface of the sample, and the height map of the surface is known directly from the deflection of the cantilever. In dynamic mode, the cantilever vibrates at or near its fundamental or harmonic frequency, while its amplitude, phase, and resonance are related to the force between the probe and the sample (Chen et al., 2021). AFM has three common imaging modes, namely, the contact mode, the non-contact mode, and the tap mode. Tap mode is the commonly used mode, like a blind man touching an elephant, slowly stroking the surface of an object, and a three-dimensional image of its surface can be visualized. In the tap mode, the tip force can be inferred from the cantilever displacement of the atomic force microscope using Hooke’s law) Equation (1) and (Beshay et al., 2022).

(Equation 1) F = k△x

F is the force acting on the tip of the cantilever, k is the cantilever stiffness, and ▵x is the displacement of the tip. This equation allows us to obtain the force for each pixel on the surface of the object.

**Figure 5:**
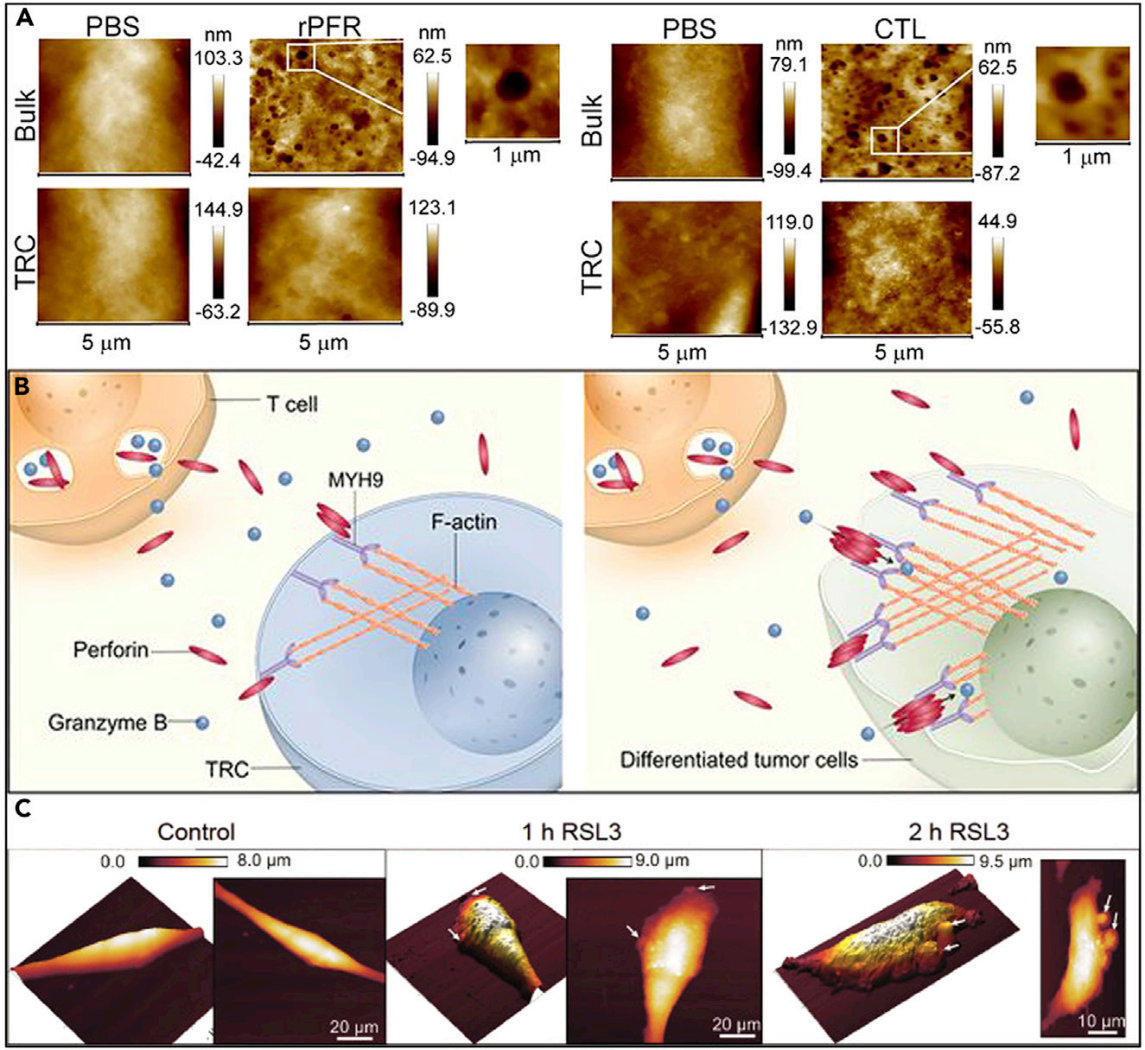
Acquisition of biomechanical signals with AFM (A) Fewer pores were observed in perforin-treated or CTL cocultured OVA-TRC compared to OVA-B16 cells (Liu et al., 2021a, 2021b, 2021c). Copyright © 2021, American Association for Cancer Research. (B) For stiff tumor cells, the perforin-MYH9 interaction generates strong phase-reversal forces, which in turn affect and alter the conformation of perforin and thus triggers subsequent chemical processes (Liu et al., 2021a, 2021b, 2021c). Copyright © 2021, American Association for Cancer Research. (C) The formation of membrane protrusions on the surface of iron-dead cells (white arrows) was characterized by AFM at different time points after stimulation of MCA205 cells with 2.5 μM RSL3 (Efimova et al., 2020). CC BY-NC 4.0.

The force information collected by AFM may respond to properties such as pressure, tension, adhesion, friction, elasticity, viscosity, and energy dissipation of an object’s surface (Qin et al., 2010). AFM can differentiate the types of substances based on their surface forces (Cross et al., 2007). For example, Wang’s group used the difference in stiffness or Young’s modulus between gold nanoclusters and nucleic acids to determine the location of gold nanoclusters in gold-nucleic acid complexes through AFM phase diagrams (Figure 5A). However, this approach is limited to nanomaterials with simple compositions, and the mechanical properties of the individual components must be very different from each other (Wang et al., 2020). Huang’s group reported using AFM to observe pores in the plasma membrane of immune cells and tumor cells. It was observed that perforin tends to form more pores in hard cells, whereas pores are difficult to form in soft cells. The study also found that MYH9 may be mechanically regulated by perforin, thereby bypassing the regulation of myosin light chain triggered by chemical signaling. This study draws an important conclusion: cellular softness is a fundamental mechanism by which tumor-regenerating cells evade T cell killing. This may provide a potential explanation for malignant cellular immune escape in patients treated with CAR-T cells or PD-1 blockade (Liu et al., 2021a, 2021b, 2021c) (Figure 5B). This brings us to the question whether immune cells other than T cells are also deformed by tumor or matrix stiffness, and whether this deformation is independent of known chemical or molecular biological signals, which requires further investigation with the aid of AFM. In addition, the same recent study found numerous uniform protrusions on the cell surface after ferroptosis in tumor cells, and these protrusions appeared to resemble the timelines of ATP and HMGB1 expressed by immunogenic cells. With the help of AFM, this is the first discovery that vaccination of early ferroptosis tumor cells may activate antitumor immunity. With the help of AFM, the affinity of immune cells for different target materials could be detected (Figure 5C). For example, Chain’s team found that the force-distance curves of AFM showed that the protein was more likely to target macrophages after being chlorinated, whereas the unmodified model protein bound to macrophages closer to background values (Prokopowicz et al., 2010).

### Magnetic resonance

In 1946, physicists discovered that atomic nuclei in a magnetic field tilt when excited by a high frequency magnetic field. After the high frequency magnetic field is turned off, the nuclei will release the absorbed energy and return to the original state, which is the theoretical basis of MRI. It was not until 1968 that Richard Ernst’s team improved the excitation pulse sequence and analysis algorithm, greatly improving the signal sensitivity and imaging speed, and MRI technology gradually matured.

**Figure 6:**
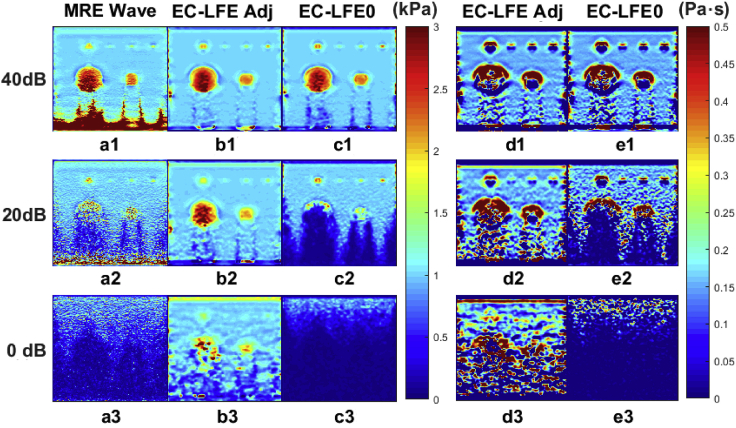
The NMR images were used to analyze the elasticity and viscosity coefficients a-c. are the elasticity images and d-e. are the medium viscosity images. The SNR magnitudes corresponding to each image row are listed on the left. Different colors are assigned to different hardness and viscosity values (Hu and Shan 2020). Copyright. © 2020 Elsevier B.V.

**Figure 7:**
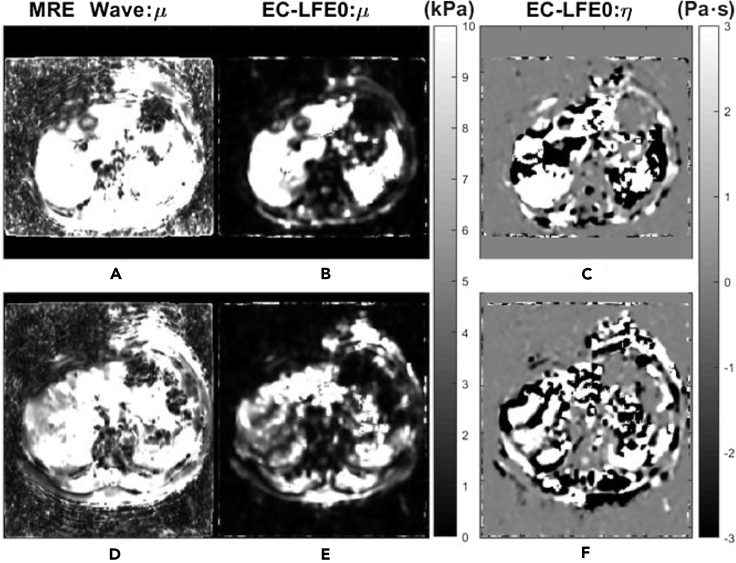
Clinical outcomes of patients with fibrosis of the liver compared with viscoelastic analysis The first and second rows show the magnetic resonance shear modulus maps of two patients with cirrhosis, respectively. A-B., D-E. are the elastograms of the patients; C,F are the viscoelastic maps of the patients. It can be seen that the first patient has lower viscoelasticity compared to the second patient. This is consistent with the clinical diagnosis that the second patient has a lower degree of fibrosis than the first patient (Hu and Shan 2020). Copyright. © 2020 Elsevier B.V.

It is well-known that with the help of magnetic resonance imaging, the size of the tumor can be determined, resulting in a medical image map (Hay et al., 2022; Florkow et al., 2022; Andescavage and Limperopoulos 2022). However, with advances in technology, there are now some instruments developed to acquire magnetic resonance mechanical signals. Magnetic resonance elastography (MRE) utilizes magnetic resonance imaging (MRI) techniques to measure the spatial distribution of mass displacement within tissue caused by external forces and as an input to solve the inverse elastodynamics problem to obtain the spatial distribution of tissue elastic coefficients (Figure 6). By collecting the elastic coefficients of different tissues or different tissue surfaces, tumor size can be estimated inversely and ultrasmall tumors can be localized (Hu and Shan 2020). In addition, techniques for *in vitro* detection of protein conformation by magnetic resonance are well established. For example, the three-dimensional structure of lactobacin A immunoprotein (LciA) can be determined by using nuclear magnetic resonance spectroscopy. The unstructured C-terminal tail was found to be important for the function of immune proteins (Kristiansen et al., 2016). The detection and analysis of the elasticity of the surrounding tissue with the aid of the detection of protein structure by magnetic resonance may help in the future to explain the mechanism of cytokines in tumor immune evasion (Figure 7).

#### Micropipette

**Figure 8:**
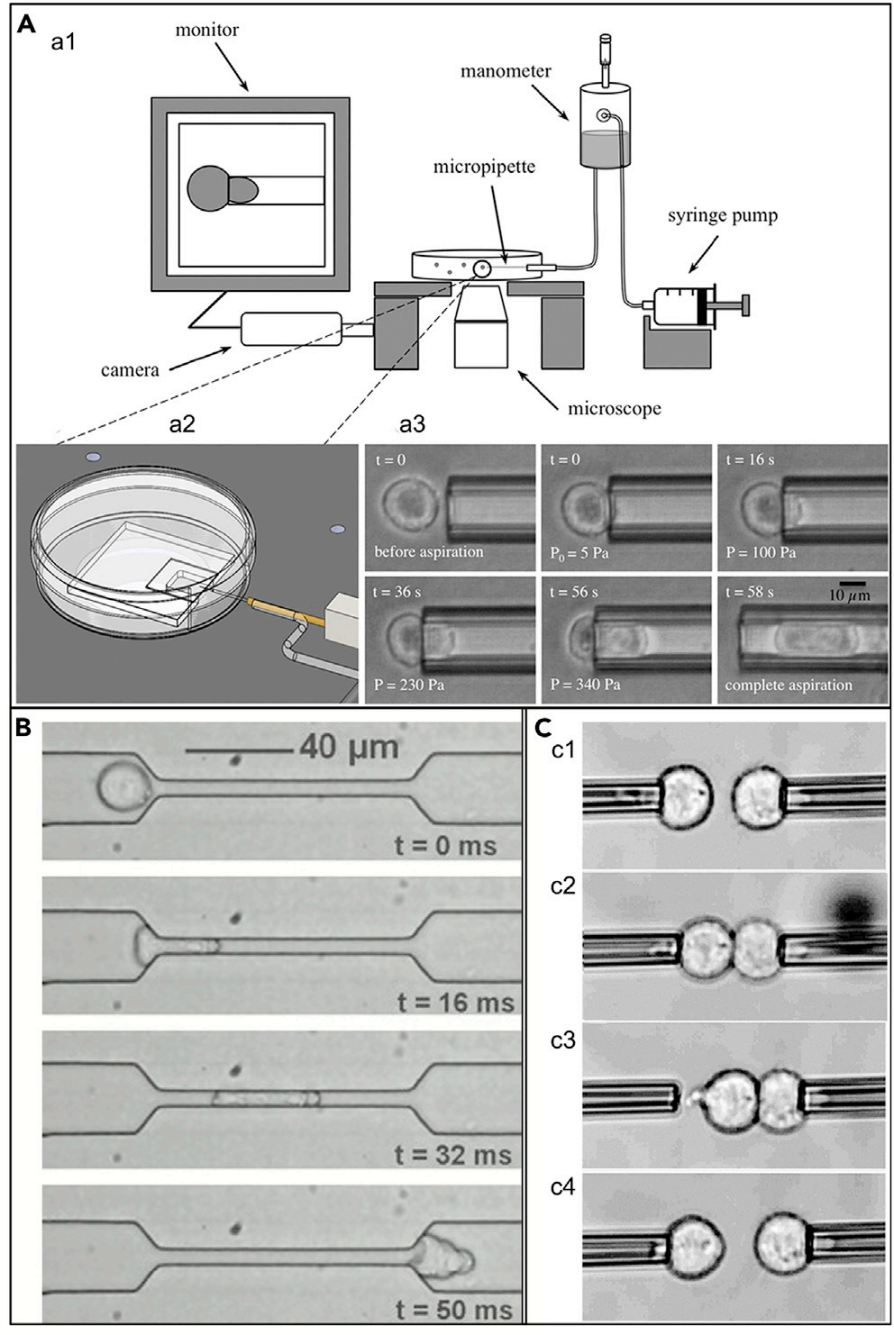
Micropipette technology (A) Schematic diagram of a typical micropipette assay (Mohammadalipour et al., 2018). Copyright 2018, John Wiley and Sons. (B) A series of deformation images of Panc-1 cancer cells as they pass through PDMS microfabricated fluid channels (Suresh, 2007). Copyright 2007, Elsevier. (C) c1.c2. is the process of contact between two cells. c3.c4. is the process of separation of the two cells after 1 s of contact (Dufour et al., 2005). Copyright 2022, American Physical Society.

Micropipettes are frequently used to examine deformability and viscoelasticity of individual cells. The main building blocks are relatively simple (shown in Figure 8A) (Beshay et al., 2022; Mohammadalipour et al., 2018). Usually an optical microscope, a microfluidic track, a small pressure pump, or a pipette are used in this method, and this method is important to detect deformability and viscoelasticity of cells (Houk et al., 2012; Esteban-Manzanares et al., 2017; Guilak et al., 2000; Nava et al., 2008). For example, cells are known to be repeatedly squeezed during systemic and pulmonary circulation. Cells that get stuck in capillaries can cause blockages that can lead to blood vessel ischemia. The physiological process of leukocytes moving in and out of tiny capillaries can be simulated with a micropipette. Initially, algorithms based on this approach simply recorded cells as spheres or viscous droplets, leading to many conclusions that did not fit human physiological parameters. Now, the model has been gradually improved on the basis of mathematical functions of the nucleoplasmic distribution, cell surface tension, droplet radius, viscosity, suction, and pipette radius. For example, Fan's team’s microfluidic chip can simulate the microenvironment of the initial stage of angiogenesis *in vitro*, and can precisely regulate the wall shear, transendothelial flow, interstitial flow, and growth factor concentration gradient of endothelial cells (Zhao et al., 2021).

As measured by this method, tumor cells were found to have greater changes in cell mechanics after keratin reorganization and were also more easily deformed when passing through areas of limited area (Suresh, 2007) (Figure 8B). At the same time, its deformability was also affected by lysophosphatidic acid. Adhesion is a more complex process than cell deformation. However, theoretical models can still be established through physical mechanics and mathematical methods. Although it is not possible to fully simulate the physiological state, it is useful to compare adhesion between different cells or between different drugs. When two deformable objects stick together, applying an external force to separate them results in deformation of their contact area. According to the degree of deformation and the strength of the external force, it can be determined according to the Johnson-Kendall-Roberts (JKR) theory. Figure 8C shows the process from adhesion to separation between two cells. The adhesion energy between mouse sarcoma cells was quantified by the strong adhesion force generated by high-concentration dextran solution (Chu et al., 2005). Currently, there is no theory based on the adhesion between immune cells and tumor cells to calculate the adhesion force under different conditions. However, quantifying the adhesion of different adhesion molecules to different cancer cells and different immune cells will surely promote the development of precision cancer therapy and genetic engineering.

#### Traction force microscope

**Figure 9:**
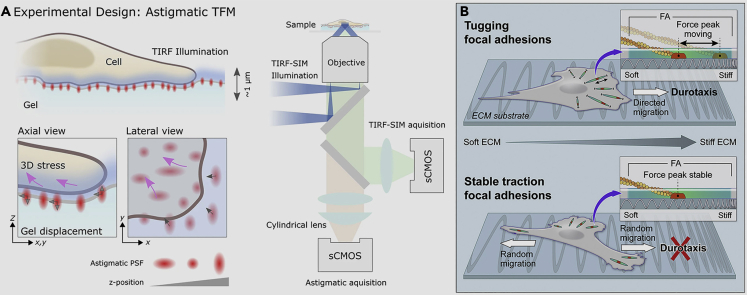
The principle of astigmatic TFM detection, and examples of TFM test (A) Diffuse imaging can be used to quantify the 3D position of the emitter with respect to the focal plane. A cylindrical lens is introduced directly in front of the camera in the optical path of the emitter, and the PSF of the microscope is reshaped according to its position relative to the focal plane. It is worth mentioning that the introduction of astigmatism allows us to extrapolate 3D information from a single wide-field image to a region of about 1 μm around the focal plane, thus eliminating the need for axial scanning (Li et al., 2021a, 2021b). Copyright CC BY4.0. (B) The traction force applied to the ECM by the adherent spot shows nanoscale variations (Plotnikov et al., 2012). Copyright 2021, Elsevier.

In the 1990s, scientists developed the first traction force microscope (TFM) (Harris et al., 1980). Since then, researchers have continued to improve it, developing various traction microscopes suitable for studying biomechanical signals on the cell surface (Liu et al., 2021a, 2021b, 2021c; Doyle et al., 2015). The working principle of astigmatic TFM is shown in Figure 9A (Barbieri et al., 2021, Li et al., 2021a, 2021b). TFM microscopy can quantitatively measure the force between cells and the matrix (Li et al., 2017). Specifically, by adding fluorescent spheres to the gel material of the matrix, the force acting on each part of the cell can be quantitatively calculated by capturing the trajectory of each fluorescent sphere as the cells move on top of the gel material. Claire-Waterman’s team increased the number of spheroids to obtain high-resolution images because of cell movement (Figure 9B) (Plotnikov et al., 2012). However, this method can only obtain the force on the interface between the cell and the matrix and cannot detect whether the cell is subjected to an upward rebound force. To overcome this shortcoming, Fabry’s team built a three-dimensional collagen model that it used to track the force of the cells (Steinwachs et al., 2016). By tracking cellular forces, the team could detect the shape, speed, and direction of breast cancer cells. Christopher Chen’s team read cell forces directly through polymer probes that support the cells like a toothbrush (Tan et al., 2003). Grashoff’s team designed a molecular sensor based on fluorescence resonance energy transfer that is more responsive to changes in small-scale forces on the cell surface (Grashoff et al., 2010). Last year, Dong Li’s team and Marco Fritzsche’s team collaborated to develop the first-generation 3D traction force microscope, which can obtain the 3D position coordinates of microspheres around cells through multilayer scanning (Li et al., 2021a, 2021b). Based on the design principles of each generation of traction force microscopes, advances in instrumentation have inevitably driven the development of cell mechanics.

The change of cell surface force seems to be like a switch, which can directly trigger the changes of intracellular proteins and signal pathways. This is of great significance to explore the immune mechanism. Wanli Liu’s team and Chunyang Xiong’s team jointly discussed the detailed characteristics and related mechanisms of immune synaptic traction in the process of B cell activation (Wang et al., 2018). By establishing a traction microscope platform, combined with a confocal live cell imaging system, the team found that B cells produce 10–20 nN of centripetal traction within 5 min from rest to activation. This traction force is distributed around the cell and can last for 30 min. *In vitro* genetic screening and rescue experiments revealed the role of some signal molecules in the proximal membrane BCR pathway in the generation and maintenance of traction. This is a great contribution to the study of B cell immune activation.

**Figure 10:**
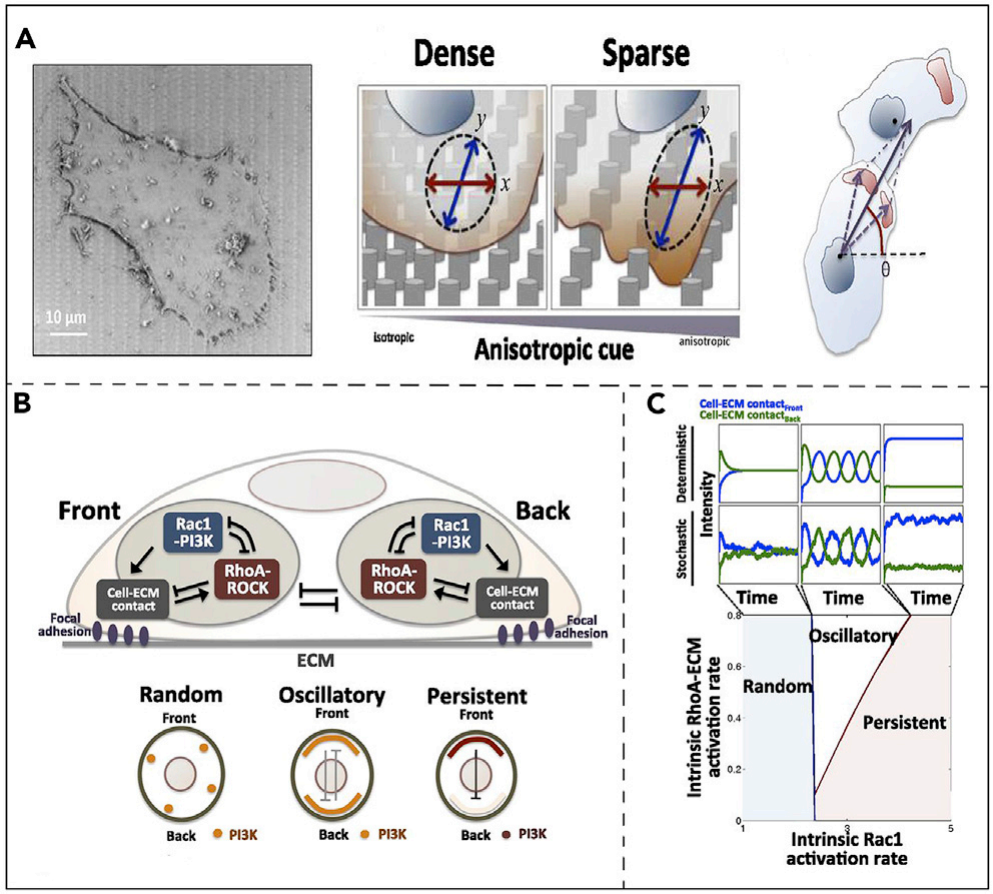
Cell polarity was analyzed by means of nanopillars on the cell surface (A) The principle of the assay is reflected in Fig. (A). (B) The signaling pathway diagram and mathematical model shown in Fig. (B). These can explain the cell polarity process. Rac1-PI3K can cellular shape shift; RhoA-ROCK can induce cell contraction. These two pathways work together to regulate cell polarity. (C) Mathematical model diagram of the different parts of cell polarity as it occurs.  (Park et al., 2017) Copyright. CC BY license.

In addition, magnetic tweezers, photoacoustic tweezers, and optical tweezers combined with optical imaging can also quantify the mechanical characteristics of tumor cells (Ashkin et al., 1986; Strick et al., 1996; Basoli et al., 2018). In one study, beads with a diameter of about 700 nm were introduced into bladder cancer cells, and the trajectory of the magnetic beads within the cells could be controlled by applying an external magnetic field (Wang et al., 2019). After following the nuclear deformation by confocal microscopy, it was found that the deformation of its long axis was smaller than that of the short axis. It can be concluded that the long axis of the nucleus is stiffer than the short axis. This may be related to the polarity of the cell, which can determine the direction of migration of the cell. It can also be used to study the effect of relaxin D and nocodazole on the polarity of the cells (Figure 10) (Park et al., 2017). In addition, the magnetic beads can be used for optical imaging of cancer cells after they have been labeled. With magnetic tweezers, it was found that cells exhibiting tumor characteristics first become soft and that the stiffness of the cells plays a major role in the cell invasion process (Swaminathan et al., 2011). Optical tweezers manipulate cells by changing the focal position through the interaction of the laser beam with microbeads. The microbeads capture the cells at the focal point of the laser beam. Following force calibration, the isoelectricity on the cell can be measured by the optical tweezers. Acoustic tweezers are used to capture and manipulate cells through the focal point of the acoustic beam (Ashkin et al., 1986; Wu 1991; Wu and Du 1990). Acoustic tweezers use lower power than optical tweezers and therefore do not affect the activity of the cells, also making their detection results potentially closer to the physiological state of the cells (Basoli et al., 2018). By looking at the stiffness and deformability of cancer cells with acoustic tweezers, it was found that the more aggressive the cancer cells were, the more deformable they were. In addition to tweezers, it is worth mentioning that many imaging techniques with the help of microscopes can also be used to detect parameters in the field of biophysics, such as photobleaching experiments. Photobleaching is (often also called fading) a phenomenon that occurs when a fluorophore loses its ability to fluoresce because of photon-induced chemical damage and covalent modifications. Fluorescence recovery after photobleaching (FRAP) can be used to characterize the flow rate distribution of a microfluid (Bonvin et al., 2010; Lusi et al., 2019).

## Future perspectives

“A life process is not just a biochemical signaling pathway.” Beth Pruitt, a mechanics engineer at Stanford University, says, “When you pull a protein, you may have to turn a binding site on or off, and it’s a cell that pulls the switch to choose which process to do.” When cells sense signals from their surroundings, it is possible that they are not regulated by the molecular pathways we know but by forces. The forces are then transduced by membrane surface proteins, which convert the forces into signals. Therefore, probing biomechanical signals within cells could help to make key advances in understanding the role of mechanics in morphogenesis, homeostasis *in vivo* and in diseases such as tumors. Tumor immune nodes, led by PD1 and PDL1, have been extensively studied. However, the clinical approach to tumor immune node therapy does not work for every patient. The reason for this, apart from the heterogeneity of the tumor, could also be that the cellular physics of its impact has not been fully explored. Biophysical research in tumor immunity could focus on Tumor-Associated Macrophages, Treg cells, and Myeloid-Derived suppressor cells; these three cells have been recognized as traitors to the immune system and can help tumor cells to immune escape. If these immune cells could be “persuaded” to surrender, it would be of great benefit to the immune cells. It would be very beneficial for tumor immunotherapy if molecular biology and biophysics could be more closely integrated and cross-checked. Naturally, biophysics has its own limitations in terms of development. In addition to the high dependence on instrumentation and the development of instrumentation, the exploration of biological information in living tissues/cells is one of the pressing issues that need to be addressed.

## Conclusion

Clinical immunotherapy methods are difficult to achieve good therapeutic effects, which may be because of the imperfect research on biophysical related mechanisms. The influence of tumor as well as matrix stiffness, fluid interstitial pressure, shear stress, and viscoelasticity on tumor immune escape is definitely present; besides, as far as the present findings show, they are mostly facilitative. The summary of biophysical research instruments will also advance the field. After studying clearly how tumors acquire biomechanical signals that help immune escape, we need to know more about how to make intervention with these biophysical signals. The development of biophysically targeted methods for tumor-related conditions may accelerate progress in this area and be useful for tumor treatment.
